# Recovering Phosphate from Complex Wastewater Using Macroporous Cryogel Composited Calcium Silicate Hydrate Nanoparticles

**DOI:** 10.3390/molecules29010228

**Published:** 2023-12-31

**Authors:** Tarawee Taweekarn, Worawit Wongniramaikul, Pariyaporn Roop-o, Wanchitra Towanlong, Aree Choodum

**Affiliations:** Integrated Science and Technology Research Center, Faculty of Technology and Environment, Prince of Songkla University, Phuket Campus, Kathu, Phuket 83120, Thailand; tarawee.t@phuket.psu.ac.th (T.T.); worawit.won@phuket.psu.ac.th (W.W.); pariyaporn.r@phuket.psu.ac.th (P.R.-o.); wanchitra.t@phuket.psu.ac.th (W.T.)

**Keywords:** starch cryogel, phosphate recovery, phosphate removal, reverse osmosis concentrate, landry wastewater, calcium silicate hydrate

## Abstract

Since currently used natural, nonrenewable phosphorus resources are estimated to be depleted in the next 30–200 years, phosphorus recovery from any phosphorus-rich residues has attracted great interest. In this study, phosphorus recovery from complex wastewater samples was investigated using continuous adsorption on cryogel column composited calcium silicate hydrate nanoparticles (CSH columns). The results showed that 99.99% of phosphate was recovered from a synthetic water sample (50 mg L^−1^) using a 5 cm CSH column with a 5 mL min^−1^ influent flow rate for 6 h while 82.82% and 97.58% of phosphate were recovered from household laundry wastewater (1.84 mg L^−1^) and reverse osmosis concentrate (26.46 mg L^−1^), respectively. The adsorption capacity decreased with an increasing flow rate but increased with increasing initial concentration and column height, and the obtained experimental data were better fitted to the Yoon–Nelson model (R^2^ = 0.7723–0.9643) than to the Adams–Bohart model (R^2^ = 0.6320–0.8899). The adsorption performance of phosphate was decreased 3.65 times in the presence of carbonate ions at a similar concentration, whereas no effect was obtained from nitrate and sulfate. The results demonstrate the potential of continuous-flow phosphate adsorption on the CSH column for the recovery of phosphate from complex wastewater samples.

## 1. Introduction

Phosphorus is an essential element in several biological molecules, including DNA, RNA, ATP, and phospholipids, that are crucial for the growth, functioning, and reproduction of all living organisms [1,2]. As phosphorus is one of the most important macronutrients needed for plant growth, it is widely used as an agricultural fertilizer and is very important for food production. Because crop production nowadays relies primarily on the application of chemical fertilizers, the usage of fertilizers has expanded dramatically and is expected to increase in the future due to the growing world population [2]. According to certain estimates, 85% of the fertilizer production market (14.2 million tons per year [3]) uses natural, nonrenewable phosphorus resources in the form of phosphate rock [2]. The finite global primary source of phosphorus is located in a few regions of the world, such as Morocco and Western Sahara [1,4,5], and estimated to be depleted in the short-to-medium term (next 30–200 years) [3,6,7,8]. Concerning global food security and the key role of phosphorus, utilizing this finite and unsubstitutable element effectively and efficiently is a global sustainability challenge. Consequently, the recovery of phosphorus from any phosphorus-rich residues has attracted great interest.

Various phosphorus-rich residues have been reported for phosphorus recovery, most of them related to the municipal wastewater treatment sector [4], including wastewater [9,10,11,12], sewage sludge [13,14], and sewage sludge char ash [15]. From the liquid phase (wastewater treatment plant influent), the phosphorus recovery rate can reach 10–60%, whereas high rates are obtained from sludge (35–70%) and sludge ashes (70–98%) [4,9]. However, phosphorus in wastewater is vital as it is partly transported to the oceans via discharge systems [9], potentially causing eutrophication and nutrient pollution. Thus, phosphorus recovery from wastewater can provide valuable organic phosphorus to agriculture production, closing the nutrient loop [4,16]. In addition, it can considerably reduce the need for chemical fertilizers, mitigate phosphorus loading on the environment, and reduce climate change impacts [16]. Moreover, phosphorus recovery from wastewater can be combined with phosphorus removal to ensure discharges with a phosphorus concentration below legislative limits [9].

The phosphorus recovery from wastewater can be achieved by applying relatively basic crystallization technologies where wastewater containing phosphorus is fed into a precipitation/crystallization tank where calcium (Ca) or magnesium salts (e.g., CaCl_2_ and Ca(OH)_2_) and seed crystals are added [9,11,12,17,18,19]. Calcium phosphate (Ca–P; CaHPO_4_·2H_2_O, Ca_8_H(PO_4_)_6_·5H_2_O, and Ca_5_(PO_4_)_3_OH) or magnesium ammonium phosphate (struvite [MgNH_4_PO_4_·6H_2_O]) then forms and is removed [20,21]. Although seeded crystallization processes for phosphorus recovery from wastewater are commercially available, such as CRYSTALACTOR^®^ and P-ROC from the Netherlands and Germany, certain factors (e.g., costs, large amounts of chemicals required, and local wastewater parameters) limit their applications. Thus, the scientific community is seeking alternative methods, especially for adsorption technology [22,23,24], aiming for simplicity, cost-effectiveness, high efficiency, and adaptable design and operation. However, the majority of publications regarding phosphate recovery through adsorption methods rely on batch adsorption studies using synthetic waters [22,25,26,27]. These studies often involve operational conditions that differ notably from real sample implementations, typically based on continuous operation systems, especially in terms of matrix composition.

In this study, phosphorus recovery was explored using continuous adsorption on a monolithic cryogel column composed of calcium silicate hydrate (CSH) nanoparticles, aiming to pave the way for future real-world applications. Initially, a standard phosphorus solution was introduced into a continuous-flow system to investigate operational parameters affecting recovery efficiency, such as flow rate, initial concentration, column height, and adsorption dynamic models. These investigations aim to facilitate the design of large-scale implementations. Subsequently, phosphorus-rich complex wastewater samples, including reverse osmosis (RO) concentrate and laundry wastewater, were introduced into the system. CSH has been investigated for decades and has reported good reactivity, settleability, filterability, dewatering, and feasibility for phosphorus recovery, where a Ca–P product contains total phosphorus (18.64%) compared with natural phosphate rock [21,28,29]. However, its practical application has been limited because of difficulty in separating fine crystallites after crystallization. The combination of CSH nanoparticles with cryogel materials can overcome this limitation, as Ca-P resulting from the interaction between phosphate and CSH active groups accumulates on porous cryogel networks [23] instead of being distributed in the liquid sample. This significantly simplifies the recovery process after the reaction, making it much easier compared to extracting fine crystallites. Additionally, as the CSH-composited monolithic cryogel column (CSH column) was applied for the continuous-flow system, results obtained from the study can facilitate a large-scale design for practical applications.

## 2. Materials and Methods

### 2.1. Materials

Disodium hydrogen phosphate (Na_2_HPO_4_) purchased from Merck (Darmstadt, Germany) was used to prepare a phosphate stock solution (100 mg L^−1^). All standard solutions and synthetic water containing phosphate were prepared by diluting a phosphate stock solution with ultrapure water generated by a water purification system (Merck, Darmstadt, Germany). Ascorbic acid (C_6_H_8_O_6_, Fisher Scientific, Leicestershire, UK), ammonium heptamolybdate ((NH_4_)_6_Mo_7_O_24_·4H_2_O, Carlo Erba, Val-de-Reuil, France), and potassium antimonyl tartrate trihydrate (C_8_H_4_K_2_O_12_Sb_2_·3H_2_O, Carlo Erba, Val-de-Reuil, France) were used for phosphate quantification using the standard ascorbic acid method [30]. Calcium chloride (CaCl_2_, 99%, Loba, Mumbai, India) and sodium silicate (Na_2_SiO_3_, Loba, Mumbai, India) were used for CSH preparation. Rice flour (Erawan Brand, Nakhon Pathom, Thailand), tapioca starch (Jaydee Brand, Nakhon Pathom, Thailand), and red lime (food-grade, purchased from a local market in Kathu, Phuket, Thailand) were used for the synthesis of the monolithic cryogel column.

### 2.2. Preparation and Characterization of the Adsorption Column

CSH nanoparticles were prepared following a previously reported rapid ultrasound-assisted sol–gel method [23,24]. A sodium silicate (Na_2_SiO_3_) solution was subjected to the gradual addition of a calcium chloride (CaCl_2_) solution under ultrasonic irradiation wherein white sol instantly appeared and gradually solidified. Further, it was taken out and allowed to gel before soaking with ultrapure water and filtering through a 0.45-μm membrane filter. The CSH nanoparticles (pore diameter: ~1.05 nm [23]) were dried in an oven before use. They were homogeneously mixed with gelatinized starch (4.5-g CSH:60-g gelatinized starch) before being filled in a plastic syringe (volume: 50 mL; diameter: 3 cm; length: 10 cm) to prepare the monolithic cryogel composited CSH nanoparticle column (CSH column) using the freeze–thaw method [23,24,31]. The obtained CSH column was removed from the container before it was cut into desired lengths (2.5, 5.0, and 7.5 cm) and put back into a plastic syringe preinstallation in a continuous-flow system.

The CSH columns pre- and postadsorption of phosphorus (phosphate form) were characterized through instrumental analysis. Their surface morphology and elemental composition were analyzed using energy-dispersive X-ray-spectroscopy (EDX)-equipped field emission scanning electron microscope (FESEM, FEI, Eindhoven, the Netherlands), whereas the functional groups of the materials were analyzed using Fourier-transform infrared spectroscopy (FTIR) equipment (Bruker, Bremen, Germany) using the attenuated total reflectance method.

### 2.3. Continuous Flow Adsorption System

A continuous-flow adsorption of phosphate on the CSH column (3 cm diameter and 10 cm length) was investigated by controlling the influent flow rate using a peristaltic pump (Gilson, Middleton, WI, USA). There was no back pressure during the operation because of the macroporous property of the monolithic cryogel column. Various parameters, including column lengths (2.5, 5.0, and 7.5 cm), initial concentrations (25, 50, and 100 mg L^−1^), and influent flow rates (2.5, 5.0, and 10 mL min^−1^) were investigated to study their influence on the recovery efficiency of phosphate using synthetic water. Effluent samples were collected from the system at time intervals before analysis for the residual concentration of phosphate using the standard spectrophotometric method [30] until the phosphate concentrations in the influent and effluent were unchanged.

### 2.4. Adsorption Data Analysis

The phosphate adsorption performance on the CSH column was investigated by plotting the breakthrough curve (BTC) for each appropriate studying parameter. A series of adsorption experimental data were analyzed, and the time-dependent ratio of the effluent phosphate concentration at time *t* to the influent phosphate concentration (*C*_t_/*C*_0_) at each studying parameter was plotted. The breakthrough time (T_b_) was defined as the time when the phosphate concentration in the effluent (*C*_t_) reached 10% of the influent concentration (*C*_0_) (*C*_t_/*C*_0_ = 0.10), whereas the exhaustion time (T_e_) was the time when *C*_t_ reached 90% of *C*_0_ (*C*_t_/*C*_0_ = 0.90) [24,32]. Adsorption parameters and adsorption dynamic models can be estimated using equations summarized in Table 1.

### 2.5. Influence of Interferences

The influence of potential interferences on the adsorption of phosphate was investigated. Potential interferences, such as sulfate (SO_4_^2−^), nitrate (NO_3_^−^), and carbonate (CO_3_^2−^) ions at 6000 mg L^−1^ [39], were mixed with 50 mg L^−1^ phosphate solution before being applied to a continuous-flow system and operated on column adsorption at optimum conditions (column height: 5 cm; influent flow rate: 5.0 mL min^−1^; temperature: 25 °C).

### 2.6. Real Sample Application

Three complex real samples containing phosphate were applied for the continuous-flow adsorption of phosphate on the CSH column. RO concentrate samples were collected from the discharge of an RO system used to recycle wastewater effluent from the Patong municipal treatment plant (Phuket, Thailand). Household laundry wastewater was collected from a laundry machine in Kathu, Phuket, Thailand. These samples were preserved in airtight polyethylene bottles and kept on ice in a styrene foam container. Synthetic laundry wastewater was prepared by dissolving detergent in tap water with the ratio recommended on the detergent packaging.

## 3. Results and Discussion

### 3.1. Characterization of Phosphate Adsorption on CSH Column

**Figure 1:**
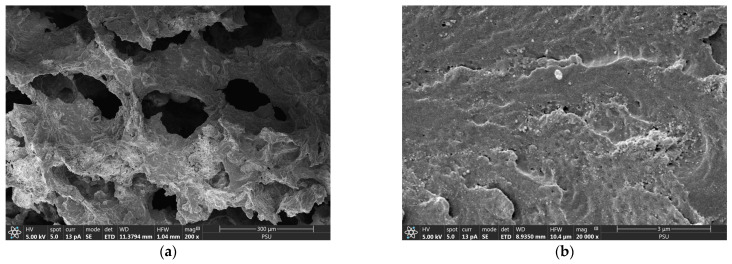
Field emission scanning electron micrographs of CSH column before phosphate adsorption with (**a**) 200× and (**b**) 20,000× magnifications.

A macroporous interconnected polymer network-like structure containing crystalline CSH nanoparticles was observed from the FESEM images of the CSH column before phosphate adsorption (Figure 1a,b). The open framework structure of the CSH column could indicate its large pore volume, potentially beneficial for phosphate access, whereas its interconnected network could facilitate influent flow through its macropores during continuous adsorption. Previous reports on the pore size distributions of CSH-composited cryogels, prepared using the same procedure as in this study, revealed significant pore volume within the pore width range of approximately 5 to 50 nm, falling within the mesopore range. Additionally, the presence of macropores (>50 nm) was also observed [23]. The surface area was reported to be 11.03 m^2^g−^1^, with a total pore volume of 0.003 mLg−^1^ [23].

**Figure 2:**
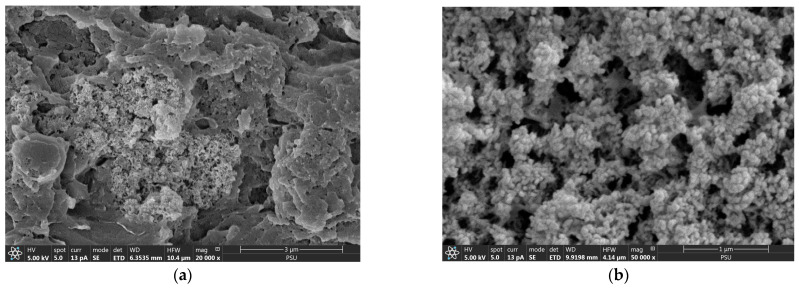
Field emission scanning electron micrographs of CSH column after phosphate adsorption with (**a**) 20,000× and (**b**) 50,000× magnifications.

After phosphate adsorption, until reaching the exhaustion time (6 h), spherical particles and their agglomeration were observed on the cryogel network (Figure 2a,b). This indicates the occurrence of amorphous Ca-P (ACP) on the CSH surface, commonly observed after a 2-hour adsorption of phosphate [20,23,24]. An ACP intermediate reportedly began transforming to hydroxyapatite (HAP) after 8 h, completing transformation 24 h postadsorption [20,23,24]. This finding confirmed that the ACP intermediate remained, without any transformation to HAP, 6 h postadsorption.

**Figure 3:**
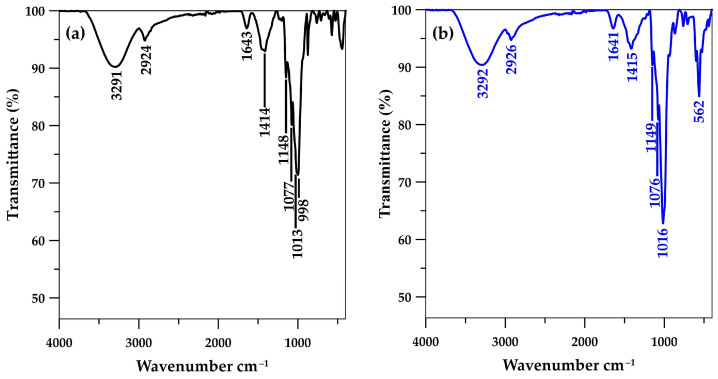
Fourier-transform infrared spectra of CSH column (**a**) before and (**b**) after phosphate adsorption.

The characterization of the CSH column prephosphate adsorption using the FTIR method revealed bands at 998 and 663 cm^−1^ (Figure 3a), indicating O–Si–O stretching and Si–O–Si bending vibrations from the unique silicon (Si)–oxygen (O) tetrahedral structure of CSH molecules [23,24,40,41] and confirming the immobilization of CSH within the cryogel network, as shown in the FESEM images (Figure 1b). The C–O–H bending of amylopectin from starch cryogel was observed at 1013 cm^−1^ in the CSH column before phosphate adsorption [31], whereas it may have overlapped with H_2_PO_4_^−^ and HPO_4_^2−^ V3 band vibration and presented as more intense peaks at 1016 cm^−1^ [20,41] postphosphate adsorption (Figure 3b). Moreover, a new intense peak of P–O vibration was observed at 562 cm^−1^, indicating phosphate adsorption on the material. These findings confirmed the presence of a Ca–P intermediate with the dominant forms of Ca(H_2_PO_4_)_2_ and CaHPO_4_ on the CSH column [28] as observed from FESEM images postphosphate adsorption for 6 h. The H–O–H bending of the water molecules in CSH [23,24] may have overlapped with the C–O bending of the amylopectin in starch cryogel [42] and presented at 1641 cm^−1^ in the CSH column preadsorption. This peak remained in its position postphosphate adsorption, similar to the O–H stretching of hydroxyl groups in the cryogel at ~3292 cm^−1^ [31,43], the C–H symmetrical scissoring of CH_2_OH moiety in amylopectin at ~1414 cm^−1^ [23], and the C–O–C vibrations in glycosidic linkage at ~1148 cm^−1^. These remaining peaks indicated no bonding/interaction between phosphate moiety and cryogel, similar to a report where CSH was immobilized on a polyvinyl alcohol sheet [41] and the phosphate recovery occurred primarily on the CSH molecules.

**Figure 4:**
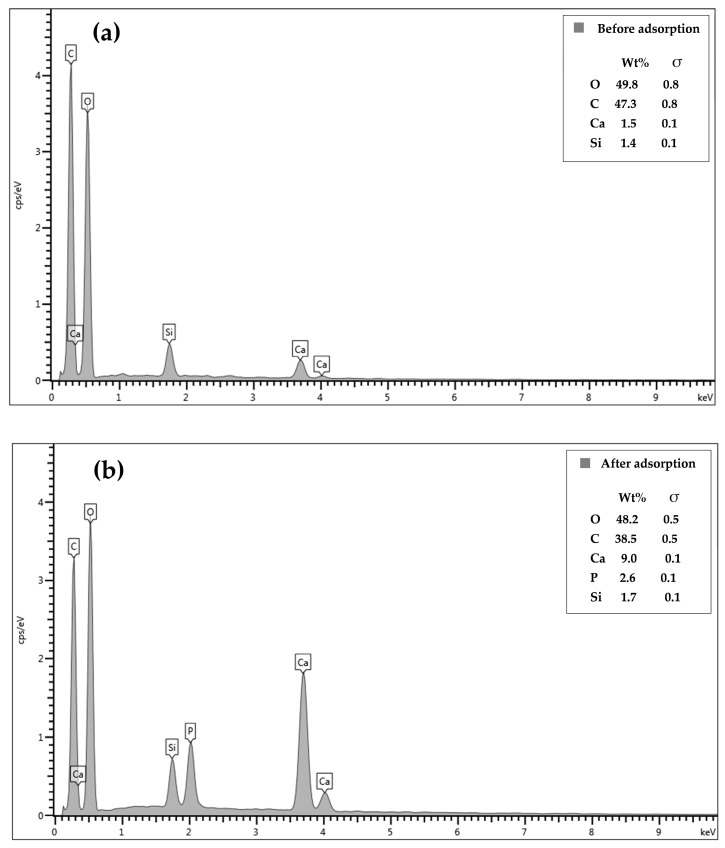
Energy-dispersive X-ray spectra of CSH column before (**a**) and after (**b**) phosphate adsorption.

The EDX results showed the presence of Ca and Si in the CSH column prephosphate adsorption, confirming the immobilization of CSH in the column (Figure 4a). The O and C contents decreased by 1.5 wt% and 8.8 wt%, respectively after phosphate adsorption (Figure 4b) whereas the Ca and Si contents increased by 7.5 wt% and 0.3 wt%, respectively. Additionally, phosphorus was found at 2.6 wt% in the material, confirming its adsorption on the CSH column.

### 3.2. Influence of Continuous-Flow Parameters on Phosphate Adsorption

#### 3.2.1. Initial Concentration

**Figure 5:**
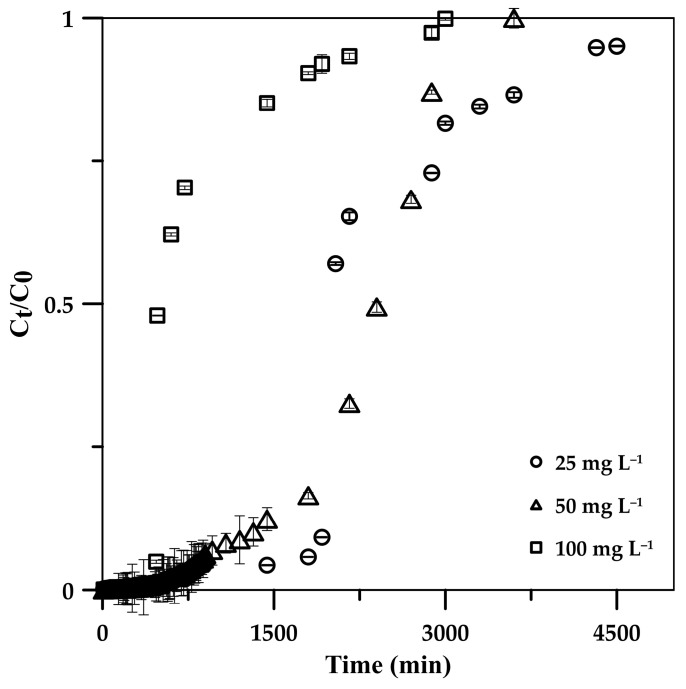
Effects of initial phosphate concentration in influent solution on phosphate adsorption breakthrough curve on 5.0 cm CSH column at 5 mL min^−1^ flow rate.

The influence of the initial phosphate concentration on the CSH column performance for phosphate adsorption was investigated by varying the influent concentration from 25 to 100 mg L^−1^ while maintaining a constant flow rate and column height of 5 mL min^−1^ and 5 cm. From the BTCs (Figure 5), it can be observed that increasing the initial concentration provided a sharp BTC, indicating a relatively small mass transfer zone at the column front when high phosphate concentration was adsorbed, potentially due to the high concentration gradient of phosphate ions on the column and in the bulk fluid and a high diffusion coefficient and mass transfer driving force at a high phosphate concentration [24,44,45]; this resulted in a great phosphate saturation rate at fixed active sites on the CSH column [46], leading to a reduction in breakthrough times (T_b_: the time at C_t_ = 0.1C_0_) from 1920 and 480 min at 25 mg L^−1^ and 100 mg L^−1^ (Table 2), whereas the exhaust time (C_t_ = 0.9C_0_) decreased from 3600 to 1920 min. The maximum capacity of phosphate (q_e_) on the CSH column increased from 115.2 to 308.5 mg g^−1^ with an increasing initial phosphate concentration from 25 to 100 mg L^−1^ due to the high mass transfer driving force [24,44,45], where the adsorption efficiency remained >99% in these concentration ranges.

#### 3.2.2. Influent Flow Rate

**Figure 6:**
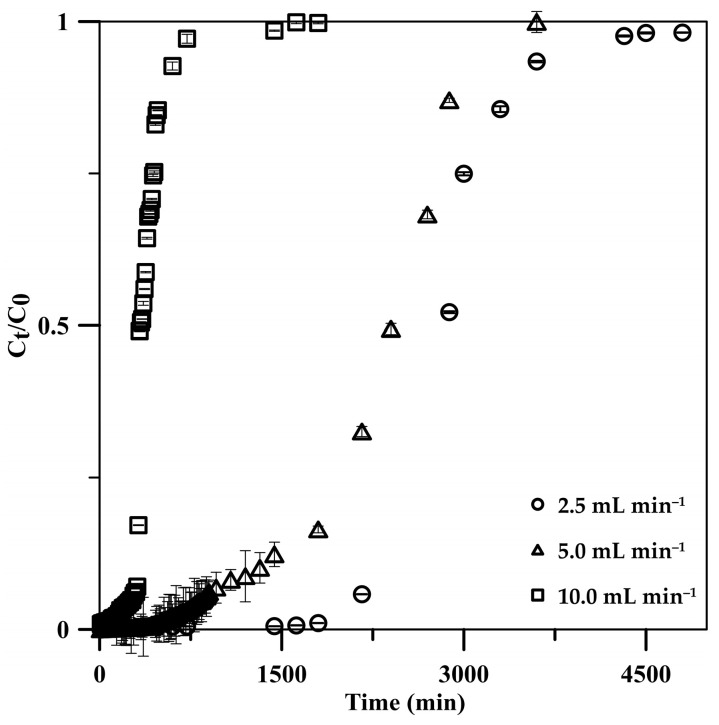
Effects of influent solution flow rate on phosphate adsorption: breakthrough curve on a 5.0 cm CSH column using a 50 mg L^−1^ phosphate standard solution.

The influent flow rate significantly influenced the adsorption performance in continuous operation; it was thus investigated in the range of 2.5–10 mL min^−1^ using a 5 cm CSH column with a 50 mg L^−1^ initial phosphate concentration. BTCs for phosphate adsorption at various flow rates displayed S-shaped curves, becoming steeper with increasing flow rates (Figure 6). The S-shaped curves depicted initial lower phosphate release into the effluent, gradually increasing until full adsorption. The rapid adsorption at the onset, across all flow rates, was due to abundant active sites on the CSH column. However, as phosphate occupied these sites, their availability diminished progressively in subsequent stages [45]. The phosphate adsorption on the CSH column reached its breakthrough faster when the flow rate was increased according to the increase in the mass transfer rate [45,46]. The breakthrough time was decreased from 2160 to 320 min, with a 2.5–10 mL min^−1^ increase in the flow rate (Table 2). However, the column could accumulate phosphate post breakthrough with low efficiency. The maximum adsorption capacity increased from 123.7 to 185.7 mg g^−1^ when the flow rate was increased from 2.5 to 5.0 mL min^−1^. Because a high number of phosphate ions were loaded into the CSH column at a 5.0 mL min^−1^ flow rate, if it reached equilibrium, high phosphate adsorption was expected on the column. The results indicated that phosphate adsorption reached equilibrium at a 5.0 mL min^−1^ flow rate, wherein phosphate ions had enough contact time on the active sites of the CSH column, potentially contributing to the macroporous structure of the monolithic cryogel. When the flow rate was further increased to 10 mL min^−1^, the adsorption capacity decreased to 165.4 mg g^−1^ due to the insufficient residence time of phosphate in the CSH column [24,45,46]. In addition, some phosphate ions probably left the column before reaching equilibrium due to stronger competition among the numerous phosphate ions to adsorb on the limited active sites in the column [24,45,46], resulting in a decrease in the adsorption efficiency from 99.99% to 99.13% with a flow rate that increased from 2.5 to 10 mL min^−1^.

#### 3.2.3. Column Height

**Figure 7:**
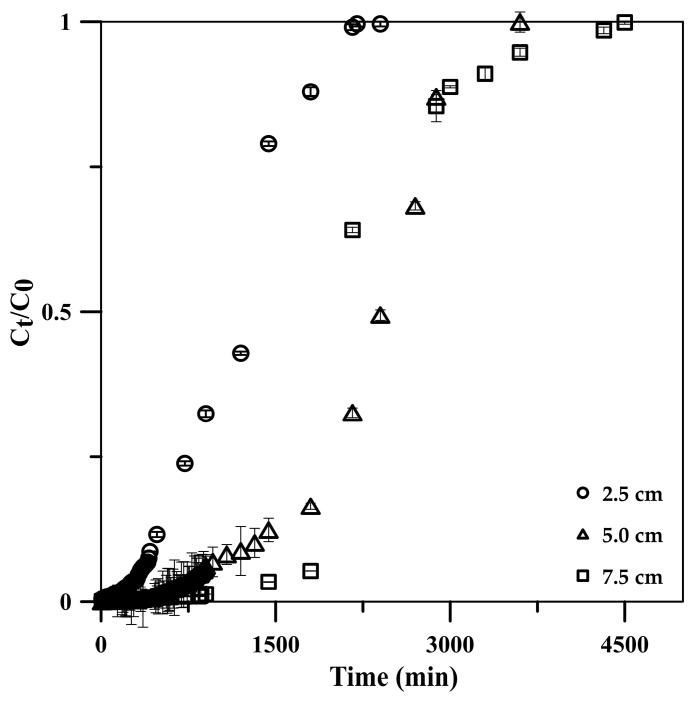
Effects of CSH column height on phosphate adsorption breakthrough curve using a 50 mg L^−1^ phosphate standard solution at 5 mL min^−1^ flow rate.

The influence of column height was investigated using the constructed BTCs at column heights of 2.5, 5.0, and 7.5 cm under a constant influent flow rate and phosphate concentration of 5 mL min^−1^ and 50 mg L^−1^, respectively. The results showed a decline in the BTC slopes (Figure 7), attributed to the long contact times between phosphate and the CSH column with a long column height [47]. The breakthrough time increased from 720 to 2160 min with the column height increasing from 2.5 to 7.5 cm (Table 2). The longer breakthrough time indicated better intraparticulate diffusion in the column, resulting in the higher adsorption capacity of the column (q_total_ increased from 599.9 to 1199.7 mg) [45,48]. Moreover, the increase in the column height increased the CSH column surface area, leading to an increase in the availability of phosphate-binding sites [24]. Additionally, the axial dispersion was decreased in the mass transfer, resulting in an increase in phosphate diffusion into the adsorbent [45,48]. Thus, phosphate had enough time to diffuse into the cryogel composite and stay longer in the column, resulting in an increased treated volume from 12,000 to 24,000 mL while maintaining a high adsorption efficiency of >99.9% at the column height range.

### 3.3. Column Adsorption Dynamics

#### 3.3.1. Adams–Bohart Model Application

The experimental data obtained from the continuous-flow operation were applied to the Adams–Bohart model [35] to study adsorption dynamics. This model assumes that the adsorption rate is proportional to the residual capacity and concentration of the adsorbate [37,49]. It is appropriate to explain the initial part of the adsorption BTC (C_t_/C_0_ is <0.5) and its linear equation as expressed in Equation (6) in Table 1. The Adams–Bohart rate constant or mass transfer coefficient (K_AB_) and saturation concentration in column (N_0_) were found to be influenced by the influent flow rate, initial concentration, and adsorbent height (Table 3), similar to previous studies [24,45,46,47,50,51]. K_AB_ increased with an increasing influent flow rate but decreased when the initial phosphate concentration and adsorbent height were increased. N_0_ decreased when the flow rate increased but was enhanced with the initial phosphate concentration and column height. The correlation coefficients (R^2^) of this model (R^2^ = 0.6320–0.8899) were similar to previous reports—R^2^ > 0.73 for azo dye adsorption onto granular activated carbon [46], R^2^ > 0.80 for methylene blue adsorption onto chitosan–clay composite [51], and R^2^ > 0.92 for phosphate adsorption on HFeO hybrid adsorbent [47]. These results indicated that external mass transfer dominated the adsorption dynamics in the initial stage [24,45,46,47,50,51].

#### 3.3.2. Yoon–Nelson Model Application

The experimental data were also applied to the Yoon–Nelson model [36] to investigate the breakthrough behavior of phosphate adsorption on the CSH column. It is a simple theoretical model, not requiring data related to the characteristics of the adsorbate, type of adsorbent, or any physical properties of the adsorption bed [24,45,46,47,50,51]. This model assumes that the decreasing adsorption rate for each adsorbate molecule is proportional to adsorbate adsorption and breakthrough on the adsorbent [24,45,46,47,50,51]. A linearized equation of the Yoon–Nelson model for a single component system is expressed as Equation (7) in Table 1, and the results are presented in Table 3. The rate constant (K_YN_) increased upon increasing the influent flow rate and phosphate concentration but decreased with the column height. These contributed to an increase in the initial phosphate concentration, increasing the competition between phosphate molecules to adsorb on active sites on the CSH column, resulting in an increased uptake rate [37,45]. The 50% breakthrough times (τ) increased with the column height but notably decreased with the influent flow rate and the initial phosphate concentration due to the faster saturation of the column [24,52].

R^2^ values of the Yoon–Nelson model were 0.7723–0.9643, which were better than those of the Adams–Bohart model (R^2^ = 0.6320–0.8899). This indicated the suitability of the Yoon–Nelson model for phosphate adsorption on the CSH column.

### 3.4. Influence of Competing Ions on Phosphate Adsorption

The influence of potential interferences (SO_4_^2−^, NO_3_^−^, and CO_3_^2−^ [39]) on the continuous-flow adsorption of phosphate was investigated, and the results are shown in Table 4. The adsorption capacity (q_total_ and q_e_) and efficiency (%RE) of phosphate showed no significant change in the presence of SO_4_^2−^ and NO_3_^−^ in the influent, although their concentrations were higher than those of phosphate by >100-fold. However, the breakthrough time significantly decreased (1320 to 440 min) when these ions were presented in the influent. Possibly, high numbers of these anions (high mass transfer driving force) could attach to CSH nanoparticles immobilized on the cryogel at the initial stage, resulting in phosphate loss in the effluent, leading to a short breakthrough time of phosphate. When the influent continuously flowed for a long time, phosphate could scramble for the binding sites back, resulting in no considerable change in other parameters. The results agree well with the batch system, reporting that the removal efficiency of phosphate remained at 99.17% ± 0.01% in the presence of mixed anions (SO_4_^2−^, NO_3_^−^, NO_2_^−^, Br^−^, and F^−^) [23]. The CSH column showed better performance for phosphate recovery than other adsorbents, such as magnesium oxide@ferric molybdate nanocomposite [39]. However, CO_3_^2−^ ions showed a notable influence on phosphate adsorption as all parameters derived from the breakthrough curves decreased in the presence of CO_3_^2−^ ions, especially at high concentration ratios. When the CO_3_^2−^ concentration was close to the phosphate concentration (1.2-fold), the phosphate adsorption performance decreased by 3.65 times.

### 3.5. Phosphate Recovery from Complex Wastewater Samples

Continuous adsorption using the CSH column was applied for the phosphate recovery from three complex wastewater samples: synthetic laundry wastewater, household laundry wastewater, and RO concentrate obtained from Patong municipal treatment plant, Phuket, Thailand. Reportedly, phosphate present in the laundry wastewater (1.84 mg L^−1^) could be recovered at 82.82%, while a high recovery efficiency of 97.58% was obtained from the RO concentrate (Table 5). The RO concentrate sample contains concentrations of 87.2 mg L^−1^ nitrate and 6.0 mg L^−1^ nitrite. Despite their presence, the high recovery efficiency of phosphate at 97.58% confirms that these common nutrient ions have minimal influence on CSH column efficiency. However, the column performance dramatically decreased when real samples were applied because of the matrix effect. Increasing the ratio of phosphate in the real sample by spiking standard phosphate substantially enhanced the column performance; for instance, q_e_ increased by 26.9 and 25.4 times for spiked RO concentrate and household laundry wastewater samples; it indicated the matrix effect playing a key role in CSH column performance, although excellent recovery efficiencies were obtained from complex wastewater samples. The continuous recovery of phosphate from the RO concentrate using the CSH column resulted in a significant 16-fold decrease in total suspended solid (TSS), consistent with findings from a prior study employing a starch cryogel column as a biofilter [31]. However, this process notably increased the chemical oxygen demand (COD) by 4.96 times and raised the total dissolved solids (TDS) by 1.7-fold. These observed increases likely stem from the release of OH^−^ and Ca^2+^ ions from the CSH [20,21,23,29].

To increase the phosphate recovery efficiency from the complex sample onto the CSH column, the operation temperature for household laundry wastewater varied in the range of 25–45 °C, improving the solution viscosity and diffusion of phosphate across the external boundary layer [32,53,54]. Increasing the temperature enhanced the maximum recovery capacity of phosphate by 57.75% (0.71–1.12 mg g^−1^) (Table 6) whereas the recovery efficiency was slightly increased from 82.82% to 86.42%. This finding indicated that increasing temperature could deplete the matrix effect.

The fully adsorbed CSH column can be easily removed from the container and utilized as fertilizer by being buried under the soil. Previous research [23] demonstrated that the material biodegrades within 10 days, implying that the phosphate present in wastewater can be reclaimed and used directly as fertilizer without generating any waste. Alternatively, the phosphate adsorbed on the CSH column can be desorbed using water [55], 1.0 M HCl [10], or 2% citric acid [55] to yield a highly concentrated phosphate solution. Subsequently, calcium-containing chemicals (e.g., CaCl_2_, Ca(OH)_2_) can be introduced to the alkaline solution to convert the phosphate into calcium phosphate fertilizer suitable for agricultural use [10]. Due to the use of starch in preparing the CSH column, it is susceptible to damage following the adsorption and desorption processes, making regeneration not advisable.

## 4. Conclusions

The simultaneous continuous recovery and removal of phosphate from complex wastewater samples was achieved using the CSH column. Phosphate in the influent was adsorbed on CSH molecules immobilized in the cryogel network, producing spherical particles of ACP and their agglomeration on the CSH column. For the synthetic 18 L water sample, 99.99% phosphate at 50 mg L^−1^ was recovered (q_total_ = 899.8 mg) via continuous adsorption on a 5 cm CSH column using a 5 mL min^−1^ influent flow rate for 6 h. The adsorption capacity of phosphate decreased with an increasing flow rate of the column but increased with increasing initial concentration and column height values, where the obtained experimental data better fitted the Yoon–Nelson model (R^2^ = 0.7723–0.9643) than the Adams–Bohart model (R^2^ = 0.6320–0.8899). Potential interferences were tested; CO_3_^2−^ ions showed a dramatic effect on phosphate adsorption, whereas no effect was observed from NO_3_^−^ and SO_4_^2−^. Phosphate in household laundry wastewater and RO concentrate (1.84–26.46 mg L^−1^) could be recovered at 82.82% and 97.58%, although a matrix effect was found. These results demonstrate the potential of continuous-flow phosphate adsorption on the CSH column for the phosphate recovery from complex wastewater samples, and the recovered phosphate, as ACP is more easily removed for further processes by taking out the CSH column postexhaust time. These findings could help facilitate the process design for industrial applications in the future.

## Figures and Tables

**Table 1:** Equations for estimating adsorption parameters and adsorption dynamics [24,32,33,34,35,36,37,38].

No.	Equation	Definition
(1)	$V_{\mathrm{eff}}=Q\cdot T_{\mathrm{total}}$	V_eff_ is the effluent volume (mL), Q is the volumetric flow rate (mL min^−1^), and T_total_ is the total flow time (min);
(2)	$q_{\mathrm{total}}=\frac{Q}{1000}\int_{t=0}^{t=\mathrm{total}}C_{\mathrm{ad}}\;\mathrm{dt}\;$	q_total_ is the total mass of adsorbed, and C_ad_ is the concentration of phosphate removal (mg L^−1^);
(3)	$q_{e\;\mathrm{column}}=\frac{q_{\mathrm{total}}}{M}$	q_e_ is the equilibrium uptake or maximum adsorption capacity at equilibrium, and M is the dry weight of adsorbent in the column (g);
(4)	$m_{\mathrm{total}}=\frac{C_{0}{\mathrm{Qt}}_{\mathrm{total}}}{1000}$	m_total_ is total number of phosphate ions entering column;
(5)	$R\%=\frac{q_{\mathrm{total}}}{m_{\mathrm{total}}}\times 100$	R% is the recovery percentage (%);
(6)	$\ln\left(\frac{c_{t}}{c_{0}}\right)={\;K}_{AB}C_{0}t-K_{AB}N_{0}\left(\frac{Z}{U}\right)$	K_AB_ (L mg^−1^ min^−1^) is the Bohart–Adams rate constant, N_0_ (mg L^−1^) is saturation concentration in column, Z (cm) is the height of monolithic column, and U (cm min^−1^) is linear rate and is calculated by dividing the flow rate (mL min^−1^) by cross sectional area of the column (cm^2^). The values of K_AB_ and N_0_ are calculated from linear plot of $\ln\frac{C_{t}}{C_{0}}$ vs. time, which are obtained from the slope and the intercept, respectively.
(7)	$\ln\frac{C_{t}}{C_{0}{-C}_{t}}=K_{\mathrm{YN}}t\;-\;\tau K_{\mathrm{YN}}$	K_YN_ is Yoon–Nelson of rate constant (min^−1^), and τ is the time required for 50% adsorbate breakthrough (min). The values of k_YN_ and τ can be estimated from the slope and the intercept from the linear plot of $\ln[C_{t}/C_{0}{\;-\;C}_{t}]$ vs. time.

**Table 2:** Parameters derived from breakthrough curves for phosphate adsorption on CSH column under various conditions.

Condition/Parameter	T_b_ * (min)	T_total_ (min)	V_eff_ (mL)	q_total_ (mg)	q_e_ (mg g^−1^)	RE (%)
Initial conc. (mg L^−1^) **						
25	1920	4500	22,500	561.6	115.2	99.84
50	1320	3600	18,000	899.8	185.7	99.98
100	480	3000	15,000	1499.2	308.5	99.94
Flow rate (mL min^−1^) ***						
2.5	2160	4800	12,000	600.0	123.7	99.99
5.0	1320	3600	18,000	899.8	185.7	99.98
10.0	320	1620	16,200	802.9	165.4	99.13
Column height (cm) ****						
2.5	720	2400	12,000	599.9	164.2	99.99
5.0	1320	3600	18,000	899.8	185.7	99.98
7.5	2160	4800	24,000	1199.7	180.7	99.98

* T_b_: the time at C_t_ = 0.1C_0_. ** 5.0 cm CSH column at 5 mL min^−1^ flow rate. *** 5.0 cm CSH column with 50 mg L^−1^ phosphate standard solution. **** 5 mL min^−1^ flow rate with 50 mg L^−1^ phosphate standard solution.

**Table 3:** Parameters derived from applying Adams–Bohart and Yoon–Nelson models to continuous-flow data.

Parameter/Model	Adams–Bohart			Yoon–Nelson		
	K_AB_ × 10^−5^ (L mg^−1^ min^−1^)	N_0_ (mg L^−1^)	R^2^	K_YN_ (min^−1^)	τ (min)	R^2^
Initial conc. (mg L^−1^)						
25	8.4	13,646	0.8899	0.0021	2669	0.9634
50	8.0	17,276	0.8543	0.0035	1870	0.8965
100	7.6	20,282	0.7994	0.0046	1443	0.8608
Flow rate (mL min^−1^)						
2.5	4.0	34,936	0.6320	0.0024	2915	0.9235
5.0	8.0	17,276	0.8543	0.0035	1870	0.8965
10	16.4	12,231	0.8599	0.0072	546	0.7723
Column height (cm)						
2.5	13.4	16,510	0.6916	0.0039	1232	0.8821
5.0	8.0	17,276	0.8543	0.0035	1870	0.8965
7.5	3.8	22,528	0.8794	0.0024	2701	0.9643

**Table 4:** Parameters derived from the breakthrough curves under various interferences.

Sample	T_b_ (min)	T_total_ (min)	V_eff_ (mL)	q_total_ (mg)	m_total_ (mg)	q_e_ (mg g^−1^)	RE (%)
PO_4_^3^^−^ (50 mg L^−1^)	1320	3600	18,000	899.8	900.0	185.0	99.99
PO_4_^3^^−^ + ^a^CO_3_^2−^	10	90	450	20.8	22.5	4.4	92.46
PO_4_^3^^−^ + ^b^CO_3_^2−^	30	180	900	43.1	45.0	9.2	96.15
PO_4_^3^^−^ + ^c^CO_3_^2−^	420	1000	5000	246.4	250.0	52.4	98.57
PO_4_^3^^−^ + SO_4_^2^^−^ + ^d^NO_3_^−^	440	3600	18,000	890.8	900.0	184.45	98.99

Concentrations of CO_3_^2^^−^: ^a^ 6000 mg L^−1^, ^b^ 600 mg L^−1^, and ^c^ 60 mg L^−1^; and of ^d^ SO_4_^2−^ and NO_3_^−^: 6000 mg L^−1^.

**Table 5:** Parameters derived from breakthrough curves of complex wastewater samples operated at optimum continuous-flow conditions.

Sample	PO_4_^3−^ Conc. (mg L^−1^)	T_b_ (min)	T_total_ (min)	V_eff_ (mL)	q_total_ (mg)	q_e_ (mg g^−1^)	RE (%)
Synthetic laundry wastewater	4.01	10	200	1000	3.37	0.70	84.04
Household laundry wastewater	1.84	10	450	2250	3.42	0.71	82.82
Household + PO_4_^3−^	51.84	30	360	1800	87.42	18.02	91.74
RO concentrate	26.46	10	120	600	15.49	3.20	97.58
RO concentrate + PO_4_^3−^	51.46	540	1620	8100	404.63	86.09	99.91
PO_4_^3−^	50	1320	3600	18,000	899.8	185.0	98.99

**Table 6:** Parameters derived from the breakthrough curves of household laundry wastewater operated at various temperatures.

Temperature (°C)	T_b_ (min)	T_total_ (min)	V_eff_ (mL)	q_total_ (mg)	m_total_ (mg)	q_e_ (mg g^−1^)	RE (%)
25	10	450	2250	3.42	4.13	0.71	82.82
35	30	480	2400	5.06	6.00	1.07	84.35
45	30	480	2400	5.29	6.12	1.12	86.42

## Data Availability

All data are available from the corresponding author on reasonable request.
